# Nonlinear threshold responses of ecosystem services supply and demand to configuration mediated by composition at cluster scale in the Yellow River Basin

**DOI:** 10.1093/pnasnexus/pgag151

**Published:** 2026-05-05

**Authors:** Qindong Fan, Zhen Ren, Guojie Wei, Baoguo Liu, Chenming Zhang, Xiaoying Ping

**Affiliations:** School of Human Settlements, North China University of Water Resources and Electric Power, Zhengzhou, Henan 450046, China; School of Human Settlements, North China University of Water Resources and Electric Power, Zhengzhou, Henan 450046, China; School of Human Settlements, North China University of Water Resources and Electric Power, Zhengzhou, Henan 450046, China; Henan Urban Planning and Design Institute Co, Ltd, Zhengzhou, Henan 450044, China; College of Landscape Architecture and Art, Henan Agricultural University, Zhengzhou, Henan 450002, China; School of Human Settlements, North China University of Water Resources and Electric Power, Zhengzhou, Henan 450046, China; School of Public Administration, North China University of Water Resources and Electric Power, No. 136 Jinshui East Road, Zhengzhou, Henan 450046, China

**Keywords:** landscape composition, landscape configuration, ecosystem services, supply and demand, threshold

## Abstract

Semi-arid river basins worldwide face pervasive imbalances between ecosystem services (ES) supply and demand, although how landscape composition mediates the nonlinear, threshold responses of these imbalances to landscape configuration remains unclear. Using the Yellow River Basin as a case study, we classified landscape-composition contexts via *k*-means clustering and developed a triple-threshold identification framework integrating quadratic polynomial fitting, segmented regression, and generalized additive models. This framework was applied to quantify how landscape configuration affects the supply–demand ratio (ESDR) of water yield, carbon sequestration (CS), soil conservation, and food production (FP). We found that (i) landscape composition governs the divergence in configuration–ESDR relationships, with regulating services (e.g. CS) shifting from surpluses in vegetation-dominated clusters to deficits in urban and desert/bare-land clusters. (ii) Within a given cluster, services respond inversely to the same configuration change: in a forest-dominated cluster, ESDR_CS increased beyond an aggregation index (AI) of ∼65.24, whereas ESDR_FP decreased beyond AI ≈ 62.84. (iii) The same configuration–service relationship exhibited opposite threshold responses across clusters; for instance, beyond a critical edge density, ESDR_CS declined in a forest-dominated cluster but increased in a desert/bare-land cluster. This study elucidates composition-mediated threshold dynamics in ES supply and demand and provides a transferable analytical framework for landscape management in large semi-arid river basins.

Significance statement As a typical mid-latitude, semi-arid large-river basin, the Yellow River Basin faces a marked imbalance between the supply and demand of ecosystem services. Existing studies still do not clearly distinguish landscape composition from landscape configuration, and they pay insufficient attention to nonlinear responses and threshold effects in landscape patterns. These gaps limit practical applications in management. By comparing “configuration–supply–demand balance” relationships across clusters with different composition backgrounds, this study identifies key change intervals and turning points that may trigger pronounced shifts in supply–demand patterns. Our results provide transferable quantitative evidence for zonation and differentiated landscape regulation in large semi-arid basins.

## Introduction

Ecosystem services (ES) refer to the products (e.g. water, food, and timber) and benefits (e.g. soil and water conservation and cultural well-being) that people obtain from ecosystem structures, processes, and functions (1, 2). Against the backdrop of rapid global urbanization and fast landscape reconfiguration, imbalances between ES supply and demand are becoming increasingly evident. These imbalances directly threaten regional sustainability and human well-being. They have also become a major barrier to achieving the United Nations Sustainable Development Goals (3–5). Many studies show that landscape patterns strongly affect ES. This influence arises from the joint roles of landscape composition (the diversity and proportion of landscape types) and landscape configuration (patch spatial form, edge characteristics, and connectivity) (6–8). Through these mechanisms, landscape patterns shape the spatial transfer of ES from supply to demand and the resulting spatial patterns of supply–demand balance (9–12).

The Yellow River Basin (YRB) is a representative large-river ecosystem in the world's mid-latitude semi-arid region (13). It contains diverse landscape types, including forests, grasslands, deserts, croplands, and urban agglomerations. Its landscape structure is highly comparable to that of major river basins such as the Mississippi and the Nile (14, 15). In recent years, rapid changes in landscape patterns have stressed the supply and demand of ES. They have also posed severe challenges to ecological security and high-quality development across the basin (16).

Existing studies have systematically revealed how landscape patterns affect the supply of individual ES (17, 18). They have also identified trade-offs and synergies among multiple services (19, 20). Building on this foundation, researchers have used segmented regression (21–23), generalized additive models (GAMs) (24, 25), quadratic polynomial fitting, and constraint line methods (26–29) to further identify nonlinear responses and threshold effects of ES to landscape-pattern change.

Here, a nonlinear response means that the ES supply–demand relationship does not change linearly with landscape patterns (30). A threshold effect means that once landscape configuration reaches a critical point, the ES supply–demand state can shift structurally. For example, it may switch abruptly from surplus to deficit or change from persistent degradation to gradual recovery (31, 32). Such behavior provides early-warning signals and actionable leverage for landscape management.

However, current research still has three major limitations. First, many studies conflate landscape composition and landscape configuration. They therefore fail to clarify their hierarchical relationship, namely how landscape composition acts as a background condition that mediates the effects of landscape configuration on ES supply and demand (33, 34). Second, most studies emphasize the supply side or overall changes in service levels. In contrast, fewer quantify surplus and deficit states from a supply–demand balance perspective and evaluate their direct contributions to human well-being (35, 36). Finally, threshold identification in prior studies often relies on a single model type and a single decision criterion (37, 38), potentially limiting both the scientific robustness and the transferability of the inferred thresholds across regions and landscape-composition contexts.

To address these challenges, we focus on the YRB and propose an integrated analytical framework of “landscape composition clustering–supply–demand quantification–threshold identification.” Specifically, we (i) identify landscape clusters based on landscape-composition characteristics; (ii) quantify the supply, demand, and ES supply–demand ratio (ESDR) for key ES within each cluster and derive the comprehensive ES supply–demand ratio (CESDR); and (iii) systematically reveal nonlinear effects and threshold intervals of landscape configuration on service supply–demand ratios under contrasting composition backgrounds.

## Results

**Figure 1:**
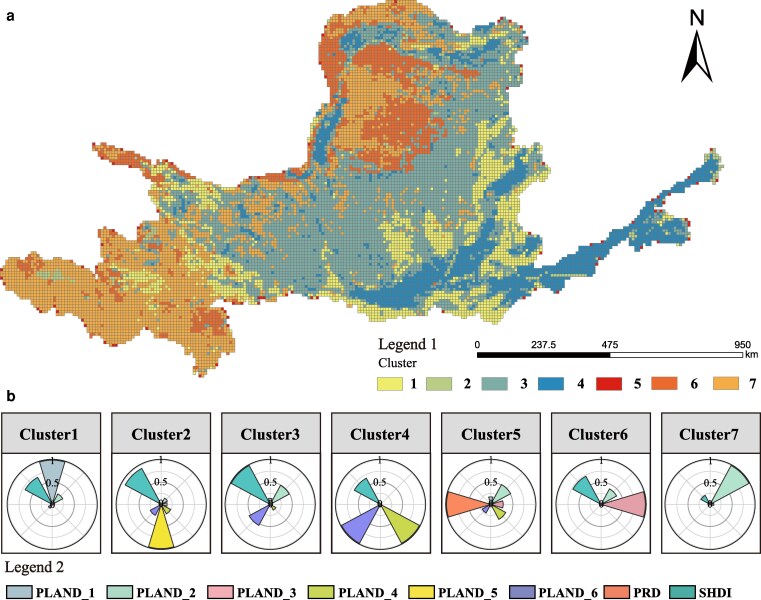
Composition-based landscape clustering patterns and comparison of cluster compositional structures in the Yellow River Basin. a) Spatial zonation of landscape clusters. b) Comparison of cluster compositional structures shown using a common radial scale.

Based on landscape-composition clustering, the YRB was classified into seven landscape-composition clusters (Fig. 1a and b): cluster 1 (forest-dominated type), cluster 2 (urban–rural transitional type), cluster 3 (grass–cultivation mixed type), cluster 4 (urban-core type), cluster 5 (multilandscape type), cluster 6 (desert/bare-land-dominated type), and cluster 7 (Steppe-dominated type). Figure 1a shows their spatial distribution, whereas Fig. 1b compares compositional structures across clusters under a common radial scale, allowing direct comparison of the same compositional variable among clusters. Mixed or mosaic clusters (e.g. clusters 2, 3, and 5) have higher compositional diversity overall, whereas single-dominant clusters (e.g. forest-, desert/bare-land-, and steppe-dominated types) show more concentrated composition structures.

These clustering results provide the basis for subsequent comparisons of the nonlinear relationships and threshold differences between landscape configuration and supply–demand balance (ESDR/CESDR) across different landscape-composition backgrounds.

**Figure 2:**
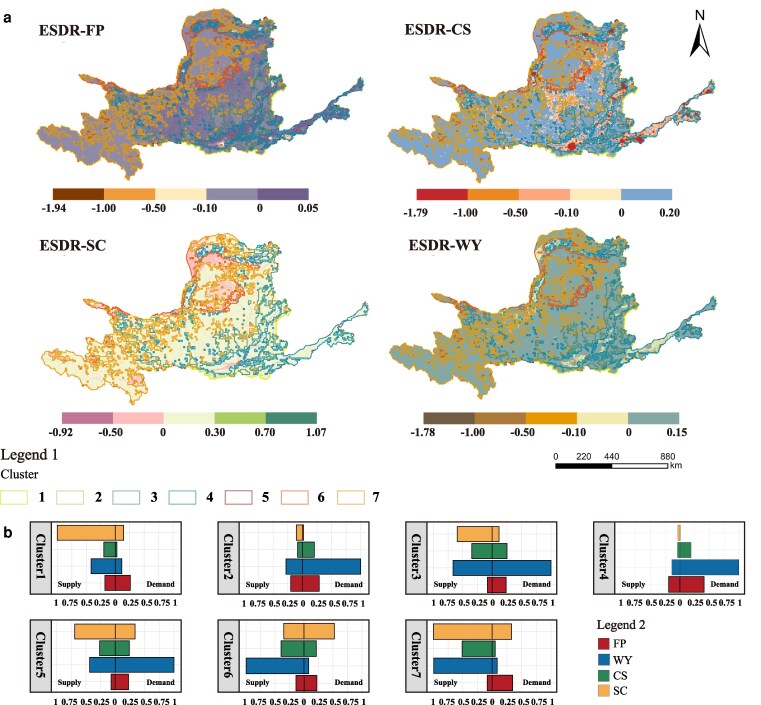
Spatial patterns of ES supply and demand and cluster-level differences in the Yellow River Basin. a) Spatial distributions of supply and demand for four ES. b) Cluster-wise comparison of ES supply versus demand.

Four ES exhibit pronounced spatial differentiation in supply–demand status across the basin (Fig. 2), as characterized by the supply–demand ratio (ESDR). Regulating services—carbon sequestration (CS) and soil conservation (SC)—more often show surpluses in clusters with higher vegetation cover but are more frequently deficit in backgrounds dominated by urban land and arid/desert or bare land. The supply–demand status of food production (FP) closely tracks cropland distribution, with surpluses concentrated in major agricultural-production clusters. Water yield (WY) displays stronger spatial heterogeneity and is closely associated with the spatial organization of the midstream valley and downstream geographic units. Cluster-level summaries further reveal distinct “service bundles” across landscape-composition backgrounds: highly urbanized or extremely arid backgrounds more often exhibit multiservice deficits, resulting in an overall lower CESDR (Fig. 2b).

**Figure 3:**
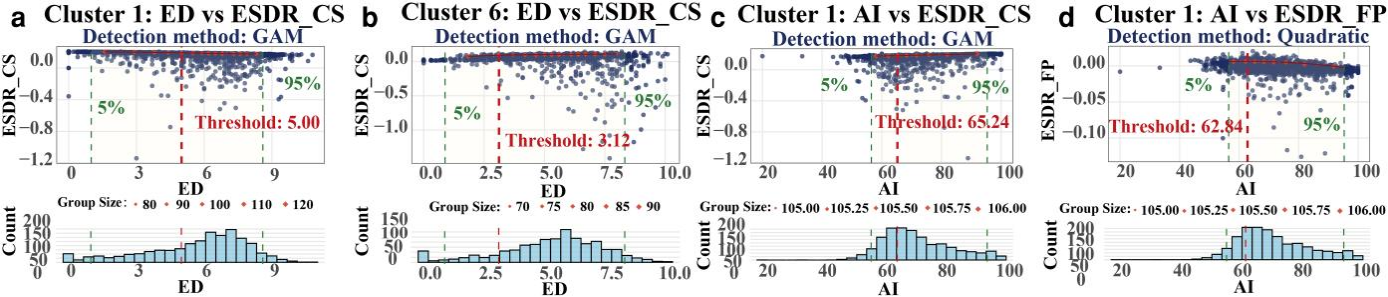
Representative within-cluster threshold relationships between landscape configuration metrics and ESDR (clusters 1 and 6). Points are observations; diamond symbols denote upper-boundary points (0.90 quantile within adaptive x-bins). Solid curves are fitted upper-boundary relationships (best-supported model; method annotated), and vertical dashed lines mark detected thresholds.

At the within-cluster scale, most “configuration metric–ESDR” relationships show nonlinear turning points. Threshold positions and postthreshold directions differ markedly across landscape-composition backgrounds. For the edge density (ED)–ESDR_CS relationship, in cluster 1, ESDR_CS decreases from 0.169 to 0.145 after ED ≈ 5.00, whereas in cluster 6, ESDR_CS increases from 0.030 to 0.035 after ED ≈ 3.12 (Fig. 3a and b; Table S13). WY shows similarly strong background dependence. In cluster 5, ESDR_WY increases from 0.017 to 0.033 after aggregation index (AI) ≈ 66.83, whereas in cluster 7, ESDR_WY decreases from 0.042 to 0.031 after AI ≈ 94.00 (Figs. S4 and S5; Table S13).

Within-cluster threshold responses further reveal pronounced cross-service differences. Cluster 1 provides a representative case: once AI exceeds the identified threshold points, ESDR increases for CS and SC, indicating strengthened surpluses, whereas FP shifts from near balance to a mild deficit. Specifically, ESDR_CS increases from 0.143 to 0.158 after AI ≈ 65.24 (Fig. 3c; Table S13), and ESDR_SC increases from 0.041 to 0.060 after AI ≈ 66.76 (Figs. S4 and S5; Table S13), whereas ESDR_FP decreases from 0.00023 to −0.00294 after AI ≈ 62.84 (Fig. 3d; Table S13).

By comparison, cluster 4 shows more consistent deficit mitigation associated with thresholds of the largest patch index (LPI). ESDR_CS increases from −0.693 to −0.299 after LPI ≈ 55.98, and ESDR_FP increases from −0.020 to −0.00576 after LPI ≈ 65.13. However, FP still declines at very high AI, with ESDR_FP decreasing from −0.010 to −0.011 after AI ≈ 76.36 (Figs. S4 and S5; Table S13).

**Figure 4:**
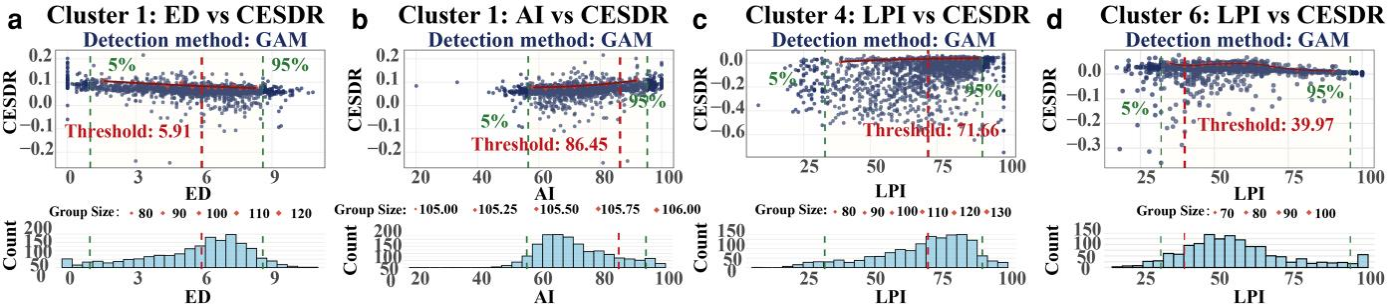
Representative intercluster threshold relationships between landscape configuration metrics and CESDR (clusters 1, 4, and 6). Plotting conventions are the same as in Fig. 3.

At the intercluster scale, CESDR thresholds further reflect differentiated shifts in overall supply–demand status across cluster backgrounds. In cluster 1, CESDR decreases from 0.072 to 0.059 after ED ≈ 5.91. It then increases from 0.062 to 0.083 when AI reaches a higher level (AI ≈ 86.45) (Fig. 4a and b; Table S14). CESDR responses to patch density (PD ≈ 0.06) and LPI (LPI ≈ 77.80) are also shown in Fig. S6 and Table S14. In cluster 4, CESDR increases from −0.139 to −0.052 after LPI ≈ 71.66, but it remains negative (Fig. 4c; Table S14). In cluster 6, CESDR increases from 0.00173 to 0.016 after LPI ≈ 40.00 (Fig. 4d; Table S14). The response at ED ≈ 4.97 and changes under very high AI (AI ≈ 76.66) are shown in Fig. S6 and Table S14.

Overall, within-cluster and intercluster results support interpreting thresholds as background-dependent-sensitive intervals rather than a single basin-wide critical value. Benjamini–Hochberg false discovery rate (BH-FDR) filtering results, model-selection summaries, uncertainty diagnostics, and the complete threshold list are shown in Figs. S4–S6 and Tables S9–S14.

## Discussion

This study demonstrates pronounced nonlinear relationships between landscape configuration metrics and ESDR and CESDR. It also identifies threshold points where service responses shift (Figs. 3 and 4; Tables S13 and S14). Previous studies have focused mainly on threshold effects in “landscape pattern–ecosystem service supply.” They often examine how metrics such as PD and LPI affect single-service supply (39, 40). In contrast, threshold features of supply–demand balance have received less attention (41). Using ESDR/CESDR as response variables, we characterize service shifts along configuration gradients within a unified framework. This approach adds evidence for how supply–demand balance changes with landscape patterns (42).

We further find systematic differences in threshold locations and postthreshold directions across landscape-composition units. The same configuration metric can even show opposite effects on the same service under different composition backgrounds (Fig. 3a and b; Table S13). For example, the relationships of ED with ESDR_CS and of AI with ESDR_WY show different threshold points and postthreshold trends across clusters. This pattern implies that treating a heterogeneous basin as a homogeneous whole, and discussing a single “universal threshold,” can obscure important spatial differences (43, 44). Compared with coarse divisions such as “urban vs. rural” (45, 46), our landscape-composition clustering delineates heterogeneous units. It therefore helps reveal finer-scale spatial differentiation in threshold effects.

These differences may reflect that ecological processes linked to landscape configuration are governed by different dominant mechanisms across backgrounds. However, our study mainly provides evidence at the level of statistical associations. Mechanistic explanations require further validation. In general, boundary- or fragmentation-related metrics, such as ED, may affect regulating services through edge effects, changes in habitat continuity, and surface processes, including runoff pathways, erosion, and material transport (47–49). In contrast, dominant-patch or aggregation-related metrics, such as LPI and AI, may influence the relative changes in provisioning and regulating services by altering vegetation continuity, connectivity, and the degree of process integration (47–49). In addition, ESDR/CESDR reflects the relative relationship between supply and demand. Postthreshold trends may therefore also be shaped by the spatial distribution of demand and by how demand couples with supply patterns (50). This provides a plausible pathway to explain why the same configuration metric can show different postthreshold directions across backgrounds.

Within clusters, services do not respond consistently to the same configuration change. Threshold points provide “locatable” structural information to describe these differences (Fig. 3c and d; Table S13). In a representative cluster, once AI crosses a threshold, ESDR for some regulating services can increase, whereas ESDR for FP may decrease. This pattern suggests that the same structural adjustment can drive opposite response directions across services. Such multiservice contrasts are widely discussed in ES research in terms of service bundles, trade-offs, and synergies (51). Threshold information helps specify the structural conditions under which these patterns emerge. Meanwhile, CESDR thresholds show that turning behavior in the composite index does not necessarily match that of any single service. Therefore, when interpreting results based on the composite index, it is important to jointly consider threshold responses of key individual services. This practice helps build a coherent chain of evidence.

Methodologically, we integrate landscape-composition clustering with a multimodel threshold identification workflow. This design reduces interpretive bias caused by mixing heterogeneous backgrounds. It also quantifies threshold uncertainty through model comparison and bootstrap resampling. We emphasize that the thresholds identified here are statistical turning points. Their ecological-process meaning should be clarified with additional process variables and scenario-based tests.

For management, our results support differentiated discussion and strategy design under landscape-composition cluster backgrounds. (i) For a single service, within each composition unit, priority should be given to configuration metrics that show clearer relationships with the target service's ESDR. Management options can then be evaluated by focusing on the gradient interval around the threshold point. (ii) For integrated goals, CESDR thresholds can inform overall assessment. At the same time, interpretations should be constrained and checked against threshold responses of key individual-service ESDR metrics. This practice helps avoid using a single composite index as a substitute for service-specific differences.

However, several limitations should be noted. First, threshold locations and postthreshold trends may depend on how ESDR/CESDR are defined and on choices of spatial resolution and aggregation units. Second, our analysis is based mainly on a single period and a static landscape pattern. It therefore cannot capture temporal dynamics in threshold behavior. Third, mechanistic explanations for multiservice contrasts require further tests using richer process variables, demand-side proxies, and scenario analyses. Future work can conduct dynamic threshold analyses using multitemporal land-use and climate data. It can also evaluate how alternative configuration adjustment pathways affect multiple services within a synergy-oriented framework.

## Conclusion

This study builds on landscape-composition clustering to identify nonlinear relationships between landscape configuration metrics and ESDR/CESDR. It also pinpoints threshold points (turning points) where ES supply–demand responses shift. Threshold locations and postthreshold directions differ markedly across composition backgrounds. At the within-cluster scale, the same configuration metric shows different turning locations and directions across services in ESDR. This pattern provides quantitative support for service-specific, differentiated configuration regulation. At the intercluster scale, CESDR threshold responses differ among clusters. This result provides quantitative reference for integrated regulation across contrasting landscape backgrounds. Notably, turning behavior in CESDR does not necessarily match that of key individual services. Integrated interpretations should therefore be assessed against threshold responses of key single-service ESDR metrics. The underlying process mechanisms and the temporal stability of these thresholds still require further testing.

## Materials and methods

### Study area and data preprocessing

**Figure 5:**
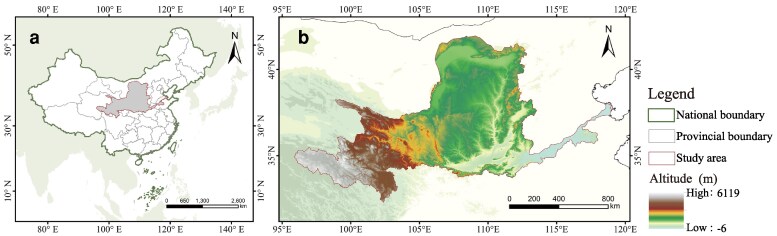
Location and topographic characteristics of the study area. a) Geographic location of the YRB. b) Extent of the study area and digital elevation model (DEM) distribution.

We used the YRB as the study area (Fig. 5) (15). The basin spans strong gradients in topography and aridity. It also shows pronounced spatial heterogeneity in land use and the intensity of human activities (52, 53). This setting is well suited to test background-dependent and threshold responses of landscape patterns in ES supply–demand relationships.

We integrated spatial datasets on land-use/land-cover (LULC), climate and topography, vegetation, and population and socioeconomic conditions to support landscape-metric calculations and estimation of ES supply–demand balance (Table S1; 2020 LULC is shown in Fig. S1). All raster layers were harmonized to a common coordinate reference system, resampled to a consistent spatial resolution, and clipped to the basin boundary; they were then subjected to consistency checks before analysis (54). To harmonize statistical and modeling scales, we first aligned all inputs to 1-km grids. We then delineated 10 km × 10 km regular grids as analysis units (*n* = 13,205) and aggregated variables to this scale using spatial aggregation and zonal statistics, ensuring that landscape metrics and supply–demand metrics were computed and aligned within the same spatial units (55, 56).

### Landscape-pattern metrics and composition-based clustering

We calculated landscape-composition and landscape configuration metrics for each grid cell. Landscape composition was characterized by patch richness density (PRD), Shannon's diversity index (SHDI), and the percentage of landscape of six land-use classes (PLAND_1–6_). Landscape configuration was characterized by PD, LPI, ED, perimeter–area fractal dimension (PAFRAC), and AI. Definitions, units, and calculation conventions for all metrics are provided in Table S2.

We used PRD, SHDI, and PLAND_1–6_ as clustering inputs and applied *z*-score standardization to each metric before clustering. We then used *k*-means clustering with Euclidean distance to delineate landscape-composition context clusters (5, 57). To improve scientific robustness, we set a random seed and repeated the algorithm with multiple random initializations of cluster centroids. We selected the number of clusters (*k*) using a two-step procedure. We first screened candidate *k* values using multiple internal diagnostic criteria. We then compared candidate solutions using internal quality metrics (mean silhouette coefficient, Dunn index, and the Calinski–Harabasz index and repeat-run consistency measured by the adjusted Rand index) (58). Based on this evaluation, we selected *k* = 7 (Tables S6 and S7). We also computed global Moran's *I* for the cluster labels to assess spatial autocorrelation. This test served as a diagnostic assessment and was not used as a constraint in the clustering procedure (Table S8) (59).

### Quantifying ES supply, demand, and supply–demand balance

We focused on key ecological challenges in the YRB, including soil erosion, carbon-sink degradation, water-resource mismatch, and food security, and selected four ES: SC, CS, WY, and FP (60). We calculated ESDR and CESDR at the grid scale and characterized supply–demand differences at both within-cluster and intercluster scales (61).

Supply was quantified as follows. SC and WY supply were estimated at the grid level using the SDR and annual water yield modules of the Integrated Valuation of Ecosystem Services and Tradeoffs (InVEST) model suite, respectively. CS supply was represented by ecosystem carbon storage derived from the InVEST Carbon Storage and Sequestration module (Tables S3–S5). Supply for FP was quantified using a linear normalized difference vegetation index (NDVI)–yield relationship, in which county-level statistical yields were downscaled to grid cells using NDVI-based weights (SI 1.3.1) (62). Demand was spatialized as follows. Demand was estimated using service-specific demand indicators (SI 1.3.2) (63). Demand for SC was represented by the difference between potential and actual erosion within the RUSLE framework. Demand for CS was estimated from population density and per-capita carbon-emission intensity. Demand for WY was allocated to corresponding land-use grid cells based on sectoral water-use quotas (domestic, agricultural, industrial, public, and ecological). Demand for FP was estimated from population density and per-capita food-consumption demand.

Indices were defined as follows. ESDR is a dimensionless index defined as the supply–demand difference after scale normalization. The normalization factor was set as the mean of the maximum supply and the maximum demand of that service across the basin; ESDR < 0 indicates a deficit, ESDR = 0 indicates balance, and ESDR > 0 indicates a surplus (SI 1.4) (64). CESDR was computed as the arithmetic mean of ESDR values across services within the same grid cell (SI 1.4) (65).

### Within-cluster and intercluster threshold identification and uncertainty assessment

Threshold identification was conducted at both within-cluster and intercluster levels using a consistent workflow. For within-cluster analyses, we treated each cluster as a grouping unit and identified thresholds for relationships between configuration metrics (PD, LPI, ED, PAFRAC, and AI) and service-specific ESDR. For intercluster analyses, we retained the same configuration metrics and used CESDR to compare integrated supply–demand threshold responses across composition backgrounds.

To reduce chance significance and spurious thresholds arising from multiple testing, we first computed Spearman correlations and applied BH–FDR correction to *P*-values (FDR < 0.05) (66). We then imposed a correlation-strength threshold and small-sample rules and fitted thresholds only for screened pairs (SI 4; Figs. S2 and S3; Tables S9 and S10).

Threshold fitting was based on upper-boundary response modeling (67). We binned the predictor across its observed range and extracted upper-boundary points of the response within each bin to form an upper-boundary point series. We then performed triple-threshold analysis by fitting three candidate models in parallel: quadratic polynomial regression, segmented regression with a single breakpoint, and GAMs. A threshold was considered valid only if it fell within the observation-supported range and key model parameter(s) met prespecified significance criteria. Model selection relied primarily on the Bayesian Information Criterion (BIC); when ΔBIC ≤ 2, we selected the less complex model based on parsimony (SI 4; Tables S11 and S12) (68). Threshold locations are reported to two decimal places in the main text, and exact estimates and CIs are provided in Tables S13 and S14.

To quantify uncertainty, we conducted bootstrap resampling for detected thresholds and reported CIs and success rates (SI 4; Tables S13 and S14).

### Software

Spatial data preprocessing, raster operations, and mapping were performed in ArcGIS 10.8. ES supply was quantified using the InVEST model suite. Landscape-pattern metrics were calculated using FRAGSTATS. Composition-based *k*-means clustering, threshold identification, and statistical analyses were conducted in R 4.4.2 with relevant add-on packages.

## Supplementary Material

pgag151_Supplementary_Data

## Data Availability

All input data used in this study are from publicly available sources, which are detailed in the Supplementary material and fully cited in the reference list. The derived data generated in this study are available in the Zenodo repository under DOI 10.5281/zenodo.18515007. All analysis codes are available in the GitHub repository at https://github.com/LandEcoResearch/es-landscape.git.
