# Supplementary material for: Nonlinear threshold responses of ecosystem services supply and demand to configuration mediated by composition at cluster scale in the Yellow River Basin
Source: PNAS Nexus. 2026 May 5;5(5):pgag151. doi: 10.1093/pnasnexus/pgag151 (PMC13176457; doi:10.1093/pnasnexus/pgag151)
Supplement: pgag151_Supplementary_Data [file pgag151_supplementary_data.pdf]

PNAS nexus

Supplementary Information for
Nonlinear threshold responses of ES supply–demand to configuration mediated by composition at cluster scale in the Yellow River Basin

Qindong Fan[1], Zhen Ren[1]*, Guojie Wei[1,2], Baoguo Liu[3], Chenming Zhang[1] , Xiaoying Ping[4]

[1]School of Human Settlements, North China University of Water Resources and Electric Power, 450046 Zhengzhou, Henan, China

[2]Henan Urban Planning and Design Institute Co, Ltd, Zhengzhou, People’s Republic of China

[3]College of Landscape Architecture and Art, Henan Agricultural University, 450002 Zhengzhou, Henan, China

[4]School of Public Administration, North China University of Water Resources and Electric Power, 450046 Zhengzhou, Henan, China

* **Corresponding authors:** Zhen Ren

Email: Z20241141080@stu.ncwu.edu.cn

Supplementary text

SI 1 Data and indicator definitions

SI 1.1 Data sources and spatial resolution

Table S1 summarizes the main input datasets used in this study, including their reference year(s) and spatial resolution. To ensure comparability across data sources and consistency in subsequent metric calculations, all spatial data were clipped and resampled under a common coordinate reference system and aligned to 1-km resolution. Climate variables were spatially interpolated from station observations to generate 1-km gridded climate layers. Socioeconomic data were downscaled to 1-km grids by integrating statistical yearbooks with proxies such as nighttime lights. We then delineated 10 km × 10 km regular grids as the basic analysis units, and performed zonal statistics/aggregation on the relevant variables at this scale, which served as the unified spatial units for landscape-pattern calculations, ecosystem service supply–demand estimation, and threshold analyses.

SI 1.2 Landscape composition and configuration metrics

To ensure consistent metric definitions, all landscape metrics were calculated within the same grid cell using a landscape-level convention and were incorporated into subsequent composition-based clustering and threshold analysis frameworks. Specifically, Patch Richness Density (PRD), Shannon's Diversity Index (SHDI), and the area proportions of six land-use classes, Percentage of Landscape ($PLAND_{1-6}$), were used to characterize landscape composition. Patch Density (PD), Largest Patch Index (LPI), Edge Density (ED), Perimeter Area Fractal Dimension (PAFRAC), and Aggregation Index (AI) were used to characterize landscape configuration and served as explanatory variables in the threshold analyses. Definitions, ecological interpretations, and calculation formulas for all metrics are provided in Table S2.

SI 1.3 Ecosystem-service supply and demand estimation

SI 1.3.1 Supply quantification

All ecosystem services (ES) supply layers were first produced at 1-km resolution and then aggregated to the 10-km analysis grid to ensure consistent spatial support across modules. Land use/land cover (LULC) classes were coded consistently across models and parameter tables (lucode 1–6: Cropland, Forest, Grassland, Water, Built-up land, and Unused land). Key biophysical parameters used to estimate soil conservation (SC), carbon sequestration (CS), and water yield (WY) supply are summarized in Tables S3–S5 (1).

(1) Soil conservation (SC)

We quantified the spatial distribution of soil retention using the Sediment Delivery Ratio (SDR) module of the Integrated Valuation of Ecosystem Services and Tradeoffs (InVEST) model suite together with the Revised Universal Soil Loss Equation (RUSLE) framework. The formulations are given in Equations 1–3, and detailed parameter definitions are provided in Table S3.

$$RKLS_x = R_x \times K_x \times LS_x \tag{1}$$

$$USLE_x = R_x \times K_x \times LS_x \times C_x \times P_x \tag{2}$$

$$SR_x = RKLS_x - USLE_x \tag{3}$$

In the formula, for grid cell x: $RKLS_x$ denotes potential soil loss, $USLE_x$ denotes actual soil loss, SR_x denotes soil retention, and R_x is the rainfall erosivity factor, K_x is the soil erodibility factor, LS_x is the slope length–steepness factor, C_x is the cover-management factor, and P_x is the support-practice factor.

(2) Carbon sequestration (CS)

We quantified the spatial distribution of carbon storage using the Carbon Storage and Sequestration module of the InVEST model suite (Equation 4). Land-cover–specific carbon pool densities are summarized in Table S4.

$$C_{total} = C_{above} + C_{below} + C_{soil} + C_{dead} \tag{4}$$

In the formula: C_{total} is total carbon storage, computed as the sum of C_{above} (aboveground biomass), C_{below} (belowground biomass), C_{soil} (soil), and C_{dead} (dead organic matter).

(3) Water yield (WY)

We quantified the spatial distribution of annual water yield using the Annual Water Yield module of InVEST (Equation 5). Parameter settings are summarized in Table S5.

$$Y_x = \left(1 - \frac{AET_x}{P_x}\right) \times P_x \quad (5)$$

In the formula: Y_x is annual water yield in grid cell x, AET_x is annual actual evapotranspiration in grid cell x, and P_x is annual precipitation in grid cell x.

(4) Food production (FP)

Following evidence that crop yield is approximately linear with the Normalized Difference Vegetation Index (NDVI), we spatialized food production by downscaling statistical grain yields with NDVI-based weights (Equation 6).

$$CP_x = \frac{NDVI_x}{NDVI_{sum}} \times CP_{sum} \quad (6)$$

In the formula: CP_x is grain yield in grid cell x, $NDVI_x$ is the NDVI value in grid cell x, $NDVI_{sum}$ is the sum of NDVI values across grid cells in the corresponding county, and CP_{sum} is the county-level statistical grain yield.

SI 1.3.2 Demand mapping

All ES demand layers were spatially mapped at 1-km resolution and then aggregated to the 10-km analysis grid to match the supply layers. Carbon sequestration demand was estimated from population density and per capita carbon-emission intensity (Equation 8). Water yield demand was allocated to relevant land-use grids by sectoral water quotas (domestic, agricultural, industrial, urban public, and ecological uses; Equation 9). Food production demand was spatialized using population density and a constant per capita annual energy intake (Equation 10).

(1) Soil conservation (SC)

The Revised Universal Soil Loss Equation (RUSLE) was used to estimate soil conservation requirements. The specific formula is given in Equation 7.

$$USLE_x = R_x \times K_x \times LS_x \times C_x \times P_x \quad (7)$$

In the formula: $USLE_x$ is actual soil loss in grid cell x, and R_x is the rainfall erosivity factor; K_x is the soil erodibility factor; LS_x is the slope length–steepness factor; C_x is the cover-management factor; and P_x is the support-practice factor.

(2) Carbon sequestration (CS)

Carbon sequestration demand at the grid scale was quantified based on the spatial distribution of per capita carbon emission intensity and population density. The specific formula is given in Equation 8.

$$D_{cx} = D_{pc} \times p_{pop} \quad (8)$$

In the formula: D_{cx} is carbon-sequestration demand ($t \cdot hm^{-2}$), D_{pc} is per-capita carbon-emission intensity ($t \cdot person^{-1}$), and p_{pop} is population density in grid cell x ($persons \cdot hm^{-2}$).

(3) Water yield (WY)

Water consumption for domestic, agricultural, industrial, and ecological purposes was allocated to the corresponding land-use grids based on sectoral water quotas. The formula used for this calculation is shown in Equation 9.

$$D_{water} = W_{dom} + W_{agr} + W_{ind} + W_{pub} + W_{eco} \quad (9)$$

In the formula: D_{water} represents water-yield demand (m^3); W_{dom}, W_{agr}, W_{ind}, W_{pub} and W_{eco} respectively represent domestic, agricultural, industrial, urban public, and ecological water use.

(4) Food production (FP)

Food production demand is primarily driven by human food requirements. Using population density, food supply demand was spatially estimated, and the calculation formula is shown in Equation 10.

$$FS_{de} = PD \times E_y \quad (10)$$

In the formula: FS_{de} denotes food-production demand, PD is population density in grid cell x, and E_y is annual per-capita food-energy intake.

SI 1.4 Definitions and calculation of ESDR and CESDR

To make supply and demand comparable across ecosystem services, we defined the ecosystem service supply–demand ratio (ESDR) as a dimensionless, standardized index (Equation 11). ESDR quantifies relative supply–demand imbalance and is scaled by the mean of the basin-wide maximum supply and maximum demand for each service. For within-cluster analyses, ESDR was computed for each service in each grid cell; ESDR < 0 indicates deficit, ESDR = 0 indicates balance, and ESDR > 0 indicates surplus. For inter-cluster comparisons, we defined the comprehensive supply–demand ratio of ESs (CESDR) as the arithmetic mean of ESDR across services within each grid cell (Equation 12)(2).

(1) Within-cluster analysis

Within each cluster, supply–demand conditions differ among individual ES. We used ESDR to characterize the supply–demand status of each ES in each grid cell (Equation 11).

$$ESDR = \frac{S-D}{(Smax+Dmax)/2} \quad (11)$$

In the formula: ESDR is the ecosystem service supply–demand ratio for a given service in grid cell x; D is demand; S is supply; $Dmax$ is the basin-wide maximum demand; $Smax$ is the basin-wide maximum supply. ESDR < 0 indicates deficit, ESDR = 0 indicates balance, and ESDR > 0 indicates surplus.

(2) Inter-cluster analysis

To characterize differences in ES supply–demand conditions across clusters, we used CESDR to represent the overall multi-service supply–demand status. CESDR was defined as the arithmetic mean of ESDR across ES types within each grid cell (Equation 12) (3).

$$CESDR_j = \frac{1}{n}\sum_{i=1}^{n} ESDR_{ij} \quad (12)$$

In the formula: $CESDR_j$ is the comprehensive supply–demand ratio of ESs in grid cell j ; n is the number of ecosystem services; $ESDR_{ij}$ is the supply–demand ratio of ES type i in grid cell j.

SI 2 Composition-based clustering and spatial coherence

SI 2.1 Clustering inputs and preprocessing

The clustering inputs include the area proportions of six land-use classes and diversity metrics (e.g., Cropland, Forest land, Grassland, Water body, Built-up land, Unused land, and Patch Richness Density (PRD) and Shannon's Diversity Index (SHDI)), used to build composition feature vectors for each grid cell. To ensure comparability across variables, all inputs were standardized using z-scores (scale()). During clustering, Euclidean distance was used without the inclusion of spatial coordinates or adjacency constraints; spatial coordinates were used only for subsequent spatial coherence testing (see SI 2.4).

SI 2.2 Choosing the number of clusters and diagnostics

Cluster number diagnosis was conducted within the range of k = 2–10, and candidate values were evaluated using Within-cluster Sum of Squares (WSS) (elbow method), Silhouette coefficient, Calinski–Harabasz (CH) index, and Gap statistics (4, 5). Gap statistics were computed using the clusGap() function with B = 100 bootstrap iterations. The Silhouette coefficient was computed using subsampling (max 2000 units) to control computational costs. The diagnostic results converged to candidate k values of 6, 7, and 10, and further comparisons were made using internal quality and stability metrics (Tables S6–S7).

SI 2.3 Cluster stability assessment

For each candidate k (6, 7, 10), we generated baseline clustering results under fixed random seeds, and repeated the clustering process under the same parameter settings to assess stability. k-means clustering was performed using the kmeans() function with nstart = 50 random initializations and a maximum number of iterations set to iter.max = 100 (6); each

k was repeated 50 times (stability_runs = 50). Stability was assessed using the Adjusted Rand Index (ARI), which measures the consistency between the baseline clustering and each repeated clustering run, with summary statistics (minimum, quartiles, and mean) provided in Table S7.

SI 2.4 Spatial autocorrelation assessment of cluster patterns (Global Moran's I)

Spatial consistency was assessed based on the final solution k = 7. A binary indicator variable was constructed for each cluster member (assigned as 1 if belonging to the cluster, 0 otherwise), and global Moran's I was calculated for each cluster (7). The spatial weight matrix was constructed based on grid cell center coordinates, creating k = 5 nearest-neighbor adjacency relationships, which were row-standardized to form the weight matrix. Moran's I was tested for significance using moran.test(..., randomisation = TRUE), and I values, z-statistics, and p-values are reported in Table S8. This test was used solely for post hoc spatial aggregation validation of clustering results and was not used as a constraint in the clustering solution.

SI 3 Candidate screening: correlation analysis with multiple-testing correction

To reduce the chance significance and spurious thresholds arising from multiple testing, threshold identification was only performed for the screened "configuration–supply/demand response" pairs. Specifically, within each cluster, Spearman's rank correlation was calculated between configuration metrics (PD, LPI, ED, PAFRAC, and AI) and supply–demand responses. For within-cluster analyses, ecosystem service-specific supply–demand responses (ESDR) were used as response variables, while for inter-cluster comparisons, composite ecosystem service supply–demand responses (CESDR) were employed. All correlation tests were two-sided. If the effective sample size was insufficient ($n < 10$) or if any variable exhibited zero variance within a cluster, correlation analysis was skipped, and the pair was excluded from subsequent threshold fitting. The Benjamini–Hochberg (BH) procedure was applied to adjust p-values for false discovery rate (FDR < 0.05) across all candidate pairs within the same cluster. Additionally, a correlation strength threshold was imposed along with small-sample rules to exclude pairs with extremely weak associations (detailed criteria are provided in Supplementary Information SI 3.1 and SI 3.2). Visualization of correlation matrices is presented in Figures S2–S3, and the list of pairs that passed screening and proceeded to threshold identification is provided in Table S9 (within-cluster ESDR analysis) and Table S10 (inter-cluster CESDR analysis).

SI 3.1 Within-cluster (ESDR): correlation tests and screening rules

Within each Cluster, we computed Spearman correlations between the five configuration metrics and the four ESDR responses. After BH–FDR control (FDR < 0.05) (8), we retained pairs with $|\rho| \geq 0.20$ and nominal $p < 0.05$. For small samples ($n < 50$), we applied a stricter rule ($|\rho| \geq 0.35$ and nominal $p < 0.10$) to improve robustness. Screened configuration–ESDR pairs used for threshold identification are summarized in Table S9.

SI 3.2 Inter-cluster (CESDR): correlation tests and screening rules

The workflow matched SI 3.1, except that the response variable was CESDR. Within each Cluster, we computed Spearman correlations between configuration metrics and CESDR, applied BH–FDR correction (FDR < 0.05), and used the same correlation-strength threshold and small-sample rules as in SI 3.1. Screened configuration–CESDR pairs used for threshold identification are listed in Table S10.

SI 4 Threshold identification and robustness assessment (within-cluster ESDR and inter-cluster CESDR)

SI 4.1 Workflow and key parameter settings (within-cluster ESDR and inter-cluster CESDR)

To reduce the influence of extreme values and sparse predictor support on threshold estimation, we applied a 5%–95% quantile constraint ($q_{0.05}$–$q_{0.95}$) to predictor X (configuration metric) before fitting. When X had few effective unique values (unique < 15), we used Winsorization to cap out-of-range values at the quantile boundaries. Otherwise, we applied trimming and retained only observations within the constrained range. Threshold

identification was not performed when the constrained sample size was $n < 30$ or when X had fewer than 6 effective unique values (9).

Threshold fitting was based on an upper-boundary point sequence. For each screened X–Y pair, we adaptively binned X with at least 5 observations per bin. Within each bin, the 0.90 quantile of Y was taken as the upper-boundary response, and the median of X was used as the representative position, yielding a sequence of (x_mid, y_upper) points. When at least five observations occurred at X = 0, we treated X = 0 as a separate bin to avoid envelope displacement caused by mixing zero and positive values. We classified support as "high" when the number of valid upper-boundary bins was ≥ 4 and as "low" when it was 3. Fewer than 3 bins were considered insufficient for threshold identification.

SI 4.2 Candidate models and selection rule (applied to within- and inter-cluster analyses)

We fitted three candidate models to the upper-boundary points in parallel: quadratic polynomial regression (Quadratic), single-breakpoint segmented regression (Segmented regression), and a generalized additive model (GAMs). Each envelope point was weighted by its bin sample size (10). All models shared a "within-support" requirement: the estimated threshold had to fall within the observation-supported range of the x_mid sequence. Thresholds outside this range were treated as extrapolation and the model was considered to have failed.

Quadratic was accepted only when the quadratic term was significant ($p \leq 0.10$) and the vertex lay within the support range. Segmented regression was optimized using three initial breakpoint values (0.3/0.5/0.7 quantiles) and required a minimum standardized slope change of 0.05 across the breakpoint to exclude near-flat pseudo-thresholds. GAMs was fitted by restricted maximum likelihood (REML) and accepted when the smooth term was significant ($p \leq 0.10$). The GAMs threshold candidate was defined as the x location with the maximum absolute second difference of the predicted curve (maximum curvature).

Model selection was based primarily on the Bayesian information criterion (BIC). When multiple models satisfied $\Delta BIC \leq 2$, we selected the simpler model by parsimony (Quadratic < Segmented < GAMs). The Akaike information criterion (AIC) was used only as a sensitivity check. Full pairwise model-comparison outputs are provided in Supplementary Data 1 (within-cluster ESDR) and Supplementary Data 2 (inter-cluster CESDR) (CSV). Cluster-level summaries are reported in Table S11 (within-cluster) and Table S12 (inter-cluster).

SI 4.3 Uncertainty and robustness criteria (applied to within- and inter-cluster analyses)

For each detected threshold (i.e., a screened and accepted X–Y pair), we performed bootstrap resampling (1,000 replicates; seed = 123). In each replicate, we repeated the same end-to-end workflow as in the main analysis (screening–upper-boundary construction–three-model fitting and selection). Bootstrap confidence intervals were computed using the percentile method (2.5%–97.5%) and were based only on successful replicates that yielded a valid threshold within the observation-supported range; replicates without a valid threshold were excluded from confidence-interval estimation. The success rate was defined as the proportion of successful replicates among all bootstrap replicates (11, 12).

To reduce the risk of chance turning points under sparse predictor support, we evaluated robustness using two diagnostics: upper-boundary support and bootstrap reproducibility. Thresholds were interpreted with caution when upper-boundary support was minimal (only three valid bins) and the bootstrap success rate was low (< 0.60). Success rates and percentile confidence intervals for all thresholds are reported in Tables S13–S14.

SI 4.4 Threshold identification curves

The plotting conventions are as follows. Points show the raw observations (x, configuration metric; y, ESDR/CESDR). Two vertical reference lines indicate the 5th–95th percentile range of x used for model fitting. Diamonds denote upper-boundary points, defined as the 0.90 quantile of y within adaptively binned x intervals; diamond size is scaled by bin sample size, and the diamond position on the x-axis is the median x value within the bin. The solid line shows the best-supported upper-boundary fit selected by model comparison, and the vertical dashed line marks the detected threshold. Histograms beneath each panel show the marginal distribution of x and include the same reference lines.

SI 4.4.1 Within-cluster threshold curves

Within-cluster configuration–ESDR threshold curves are summarized in Figs. S4–S5. These figures complement the representative examples in Fig. 3. Using the unified plotting conventions (SI 4.4), they present the remaining robust within-cluster threshold curves across multiple clusters, configuration metrics, and services.

SI 4.4.2 Inter-cluster threshold curves

Inter-cluster configuration–CESDR threshold curves are summarized in Fig. S6. This figure complements the representative inter-cluster examples in Fig. 4. Using the unified plotting conventions (SI 4.4), it presents the remaining robust CESDR threshold curves.

Figures and Tables

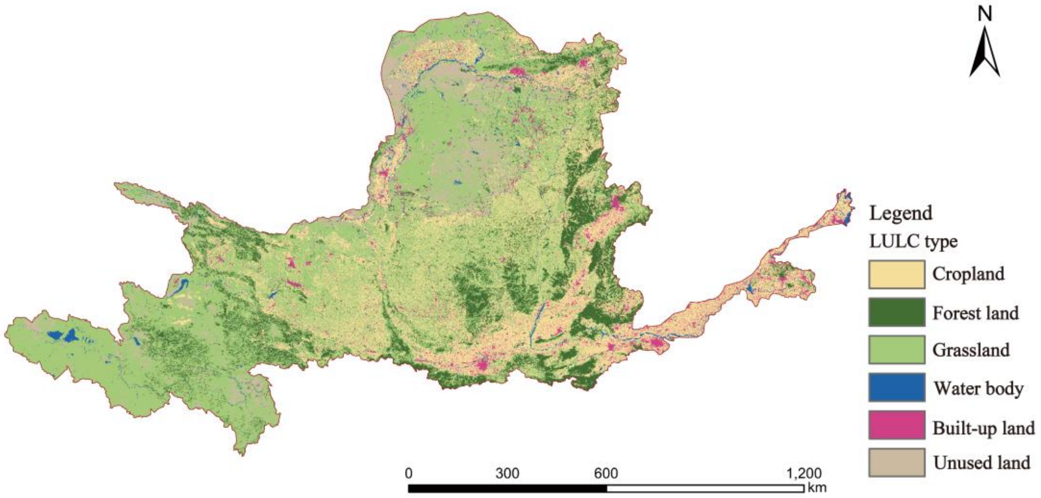

Fig. S1. Land use/land cover (LULC) distribution in the Yellow River Basin (2020).

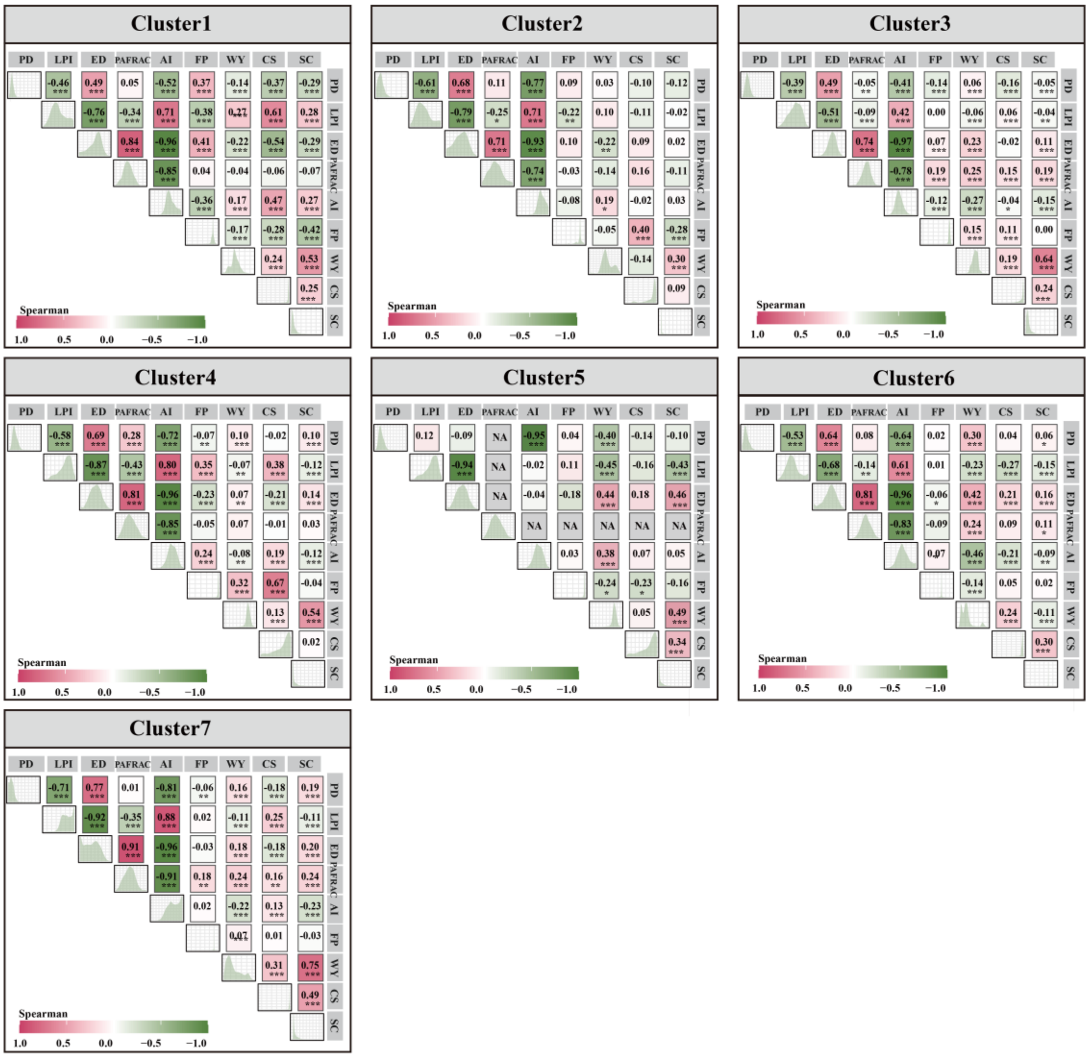

Fig. S2. Within-cluster ESDR correlation screening matrix (Spearman's ρ). Cells show the direction and strength of associations between configuration metrics and ESDR; significance is evaluated using BH–FDR correction (FDR < 0.05). Retained pairs passed to threshold identification are listed in Table S9.

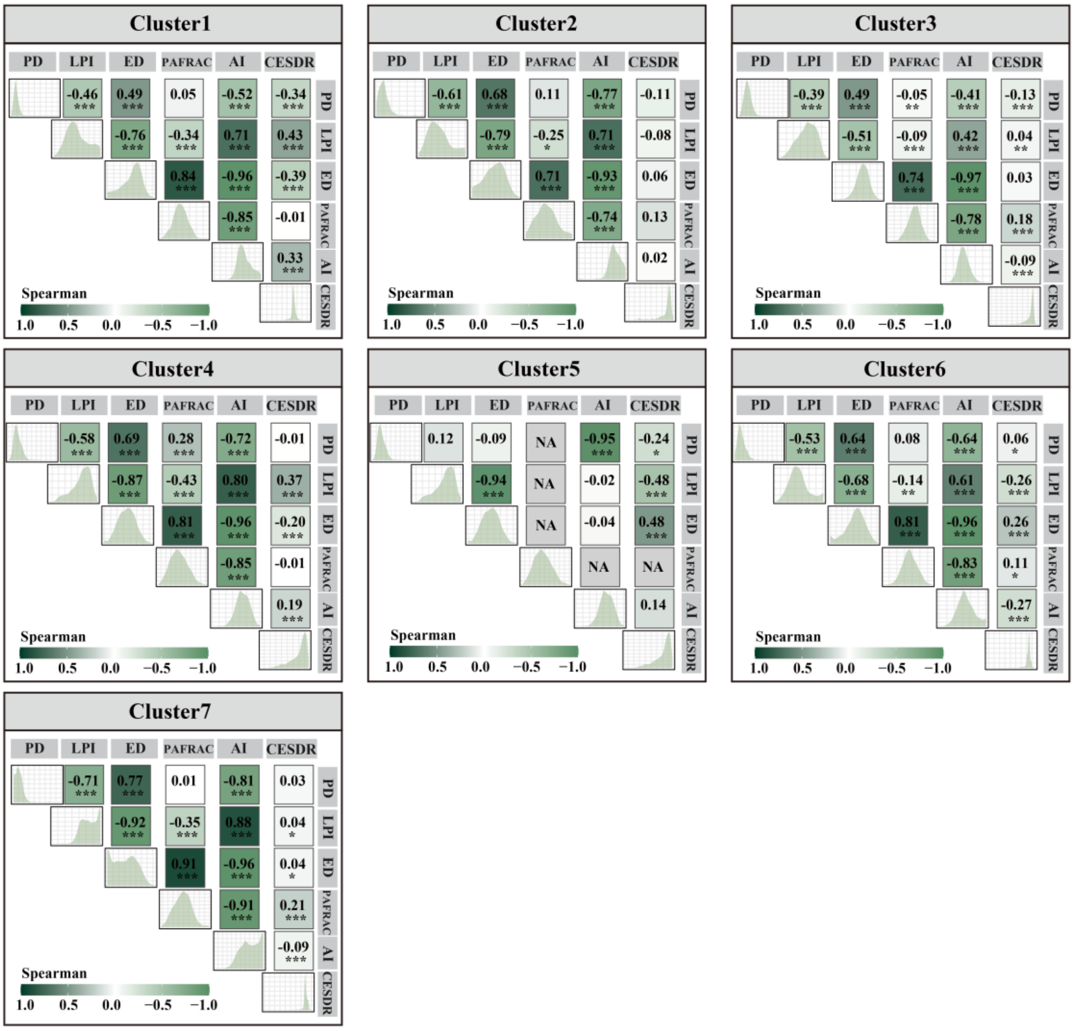

Fig. S3. Inter-cluster CESDR correlation screening matrix (Spearman's ρ). Significance is evaluated using BH–FDR correction (FDR < 0.05). Retained pairs passed to threshold identification are listed in Table S10.

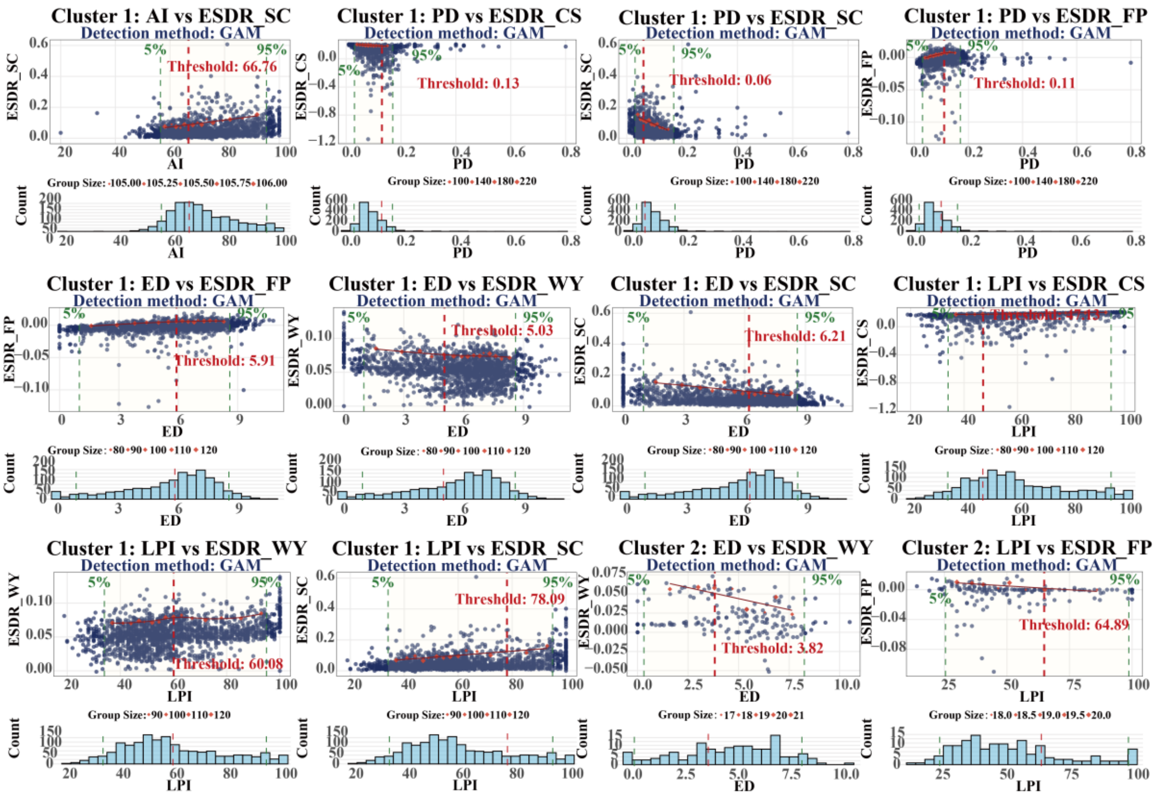

Fig. S4. Additional within-cluster threshold curves (set 1). Each panel shows the configuration–ESDR threshold pattern for a selected cluster–pair; plotting elements are defined in SI 4.4 (legend).

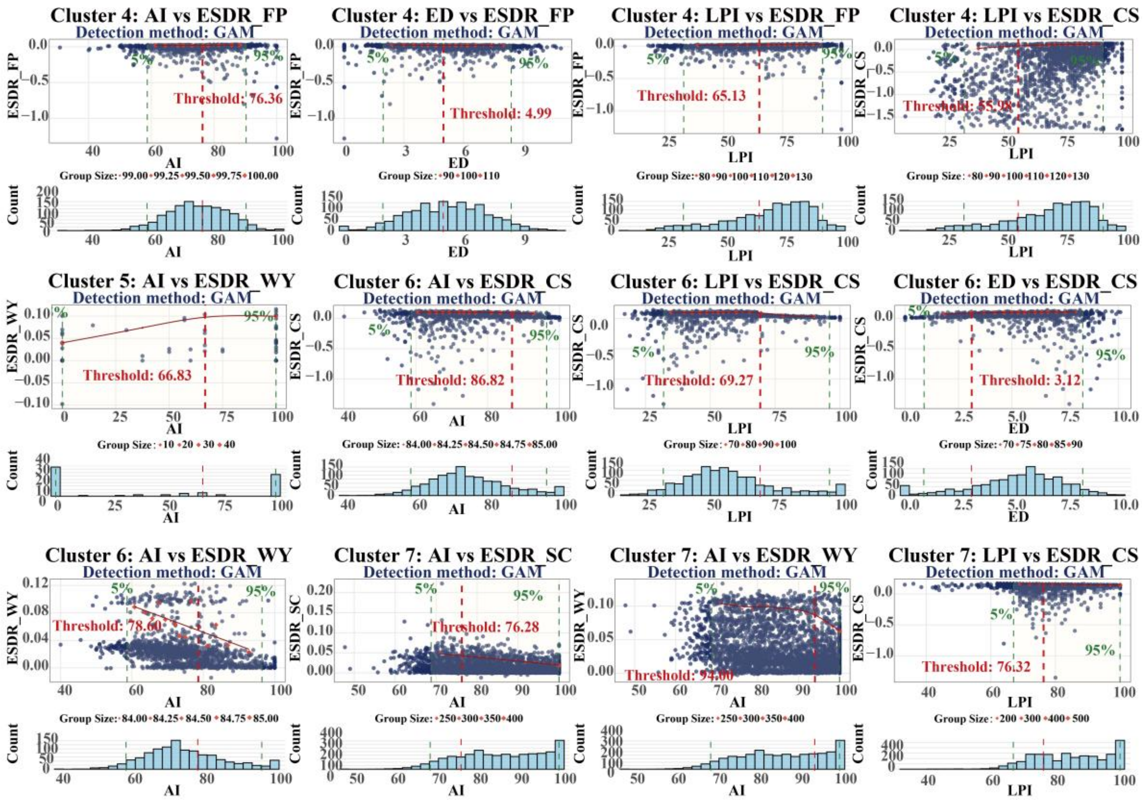

Fig. S5. Additional within-cluster threshold curves (set 2). Same as Fig. S4; see SI 4.4 for the plotting legend and parameter conventions.

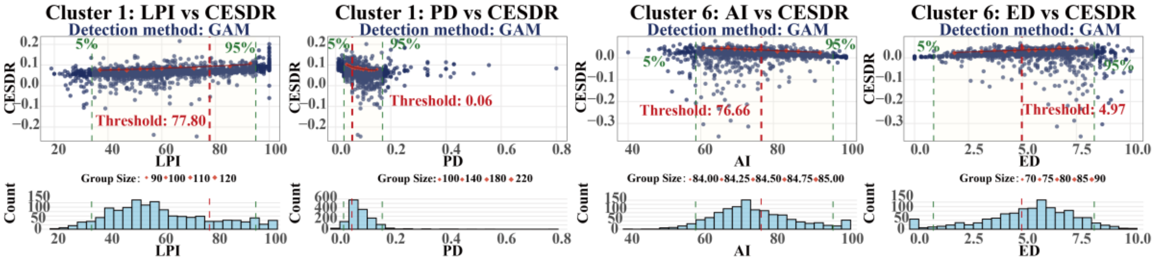

Fig. S6. Additional inter-cluster threshold curves for CESDR. Each panel shows the configuration–CESDR threshold pattern for a selected cluster; plotting elements are defined in SI 4.4 (legend).

Table S1. Data sources, years, and spatial resolution of input datasets.

Data name	Data source	Year	Spatial resolution
Basic geographic information	Data Center for Resources and Environment Science, Chinese Academy of Sciences (http://www.resdc.cn/)	—	—
Socio-economic data	Based on the 2020 statistical yearbooks of various provinces, combined with the nighttime light data of Luojia-1 (http://59.175.109.173:8888/), the data were allocated to 1 km grids using the spatial downscaling method.	2020	1km ×1 km
Land use/cover	Data Center for Resources and Environment Science, Chinese Academy of Sciences (http://www.resdc.cn/)	2020	1km ×1 km
Normalized difference vegetation index (NDVI)	Data Center for Resources and Environment Science, Chinese Academy of Sciences（https://www.resdc.cn/）	2020	1km ×1 km
Elevation data	Data Center for Resources and Environment Science, Chinese Academy of Sciences (http://www.resdc.cn/)	2020	1km ×1 km
Climate data	Using the Kriging interpolation method, the station observation data from the National Meteorological Information Center (http://www.nmic.cn/) were spatially processed.	2020	1km ×1 km
Soil data	Harmonized World Soil Database (https://www.fao.org/)	2020	1km ×1 km
Total soil water evaporation and transpiration loss	Based on MODIS NDVI data, the calculation was performed using the pixel binary model.	2020	1km ×1 km

Table S2. Definitions of landscape composition and configuration metrics used in clustering and threshold analyses.

Dimension	Indicator	Description and Ecological Significance	Computational formula
Composition	①PRD	Patch Richness Density; reflects the diversity of landscape types per unit area	$PRD = \frac{m}{A}$
	②SHDI	Shannon's Diversity Index; characterizes the evenness of the distribution of landscape patch types	$SHDI = -\sum_{i=1}^{m}(P_i \ln P_i)$
	③PLAND	Percentage of Landscape; quantifies the dominant types of landscape composition	$PLAND_k = \frac{A_k}{A} \times 100\%$
Configuration	①PD	Patch Density; indicates the degree of landscape fragmentation	$PD = \frac{N}{A}$
	②LPI	Largest Patch Index; indicates dominance of the largest patch	$LPI = \frac{\max(a_{ij})}{A} \times 100\%$
	③ED	Edge Density; characterizes edge intensity/fragmentation in the landscape mosaic	$ED = \frac{E}{A}$
	④ PAFRAC	Perimeter-Area Fractal Dimension; describes the irregularity of patch shapes	$PAFRAC = \frac{2\ln(P)}{\ln(A)}$
	⑤AI	Aggregation Index; measures the spatial aggregation of patches of the same type	$AI = \left[\frac{g_{ii}}{\max(g_{ii})}\right] \times 100\%$

Note: m is the number of land-use/cover classes; A is total landscape area; P_{i} is the proportional area of class i ; A_{k} is the area of class k; N is total number of patches; E is total edge length; P is patch perimeter; g_{ii} is the number of like adjacencies for class i ($max(g_{\mathrm{ii}})$ denotes the maximum possible like adjacencies given the composition).

Table S3. Parameters used for soil conservation (SC) estimation based on RUSLE.

Lucode	Description	usle_c	usle_p
1	Cropland	0.23	0.3
2	Forest	0.08	1
3	Grassland	0.24	1
4	Water	0	0
5	Building	0	0
6	Unused land	1	1

Note: Land-use–specific P and C factors for the Yellow River Basin (parameters for the modified Universal Soil Loss Equation; land-use codes are consistent with the land-use classification used in this study).

Table S4. Carbon pool density parameters used for carbon storage (CS) estimation.

Lucode	LULC_Name	Cabove	Cbelow	Csoil	Cdead
1	Cropland	4.94	23.45	31.49	2.84
2	Forest	12.32	33.67	46.14	4.09
3	Grassland	10.26	25.13	29.13	2.19
4	Water	0.09	0	0	0
5	Building	0.73	7.99	0	0
6	Unused land	0.38	0	6.28	0

Note: Land-use–specific carbon density values for each carbon pool in the Yellow River Basin (used in the InVEST carbon storage module; land-use codes are consistent with the land-use classification used in this study).

Table S5. Biophysical parameters used in the InVEST water yield model.

Lucode	Description	root_depth	Kc	LULC_veg
1	Cropland	2100	0.7	1
2	Forest	5200	1	1
3	Grassland	2600	0.65	1
4	Water	100	1	0
5	Building	100	0.3	0
6	Unused land	300	0.2	0

Note: Land-use–specific parameters for the InVEST water-yield module in the Yellow River Basin (land-use codes are consistent with the land-use classification used in this study).

Table S6. Cluster-number selection diagnostics across k = 6, 7, 10.

k	avg.silwidth	dunn	ch
6	0.338	0.0027	3596.1
7	0.351	0.0027	4012.3
10	0.311	0.0024	4087.3

Table S7. Cluster stability across repeated runs (Adjusted Rand Index, ARI).

k	Min	Q1	Median	Mean	Q3	Max
6	0.994	0.996	0.998	0.998	0.998	1
7	0.888	1	1	0.998	1	1
10	0.554	0.797	0.799	0.861	0.999	1

Table S8. Global Moran's I results for cluster membership indicators (post hoc spatial coherence test, k=7).

k	cluster	I	z_value	p_value
7	1	0.607	114.14	<0.001
7	2	0.338	63.71	<0.001
7	3	0.542	101.96	<0.001
7	4	0.624	117.36	<0.001
7	5	0.674	126.87	<0.001
7	6	0.033	6.2	<0.001
7	7	0.612	115.21	<0.001

Note: Spatial weights were constructed using k-nearest neighbors (k = 5) based on grid centroids and row-standardized; significance was assessed by randomization.

Table S9. Candidate configuration–ESDR pairs retained for threshold identification after Spearman screening with BH–FDR control (within-cluster).

Cluster	Response	Landscape	n	Spearman_r	p_raw	p_fdr_BH
1	ESDR_CS	AI	1759	0.465	3.33e-95	2.22e-94
1	ESDR_CS	ED	1759	-0.537	4.76e-132	4.76e-131
1	ESDR_CS	LPI	1759	0.608	1.19e-178	2.37e-177
1	ESDR_CS	PD	1759	-0.371	1.39e-58	3.97e-58
1	ESDR_FP	AI	1759	-0.358	3.15e-54	7.88e-54
1	ESDR_FP	ED	1759	0.407	2.93e-71	1.47e-70
1	ESDR_FP	LPI	1759	-0.377	1.14e-60	4.57e-60
1	ESDR_FP	PD	1759	0.374	1.27e-59	4.23e-59
1	ESDR_SC	AI	1759	0.267	3.62e-30	5.57e-30
1	ESDR_SC	ED	1759	-0.292	8.27e-36	1.84e-35
1	ESDR_SC	LPI	1759	0.284	6.18e-34	1.12e-33
1	ESDR_SC	PD	1759	-0.291	1.19e-35	2.38e-35
1	ESDR_WY	ED	1759	-0.218	2.18e-20	3.11e-20
1	ESDR_WY	LPI	1759	0.275	7.12e-32	1.19e-31
2	ESDR_FP	LPI	170	-0.218	4.22e-03	4.22e-02
2	ESDR_WY	ED	170	-0.225	3.21e-03	4.22e-02
3	ESDR_FP	PAFRAC	4740	0.204	4.69e-26	1.56e-25
3	ESDR_WY	AI	4740	-0.269	2.95e-79	5.89e-78
3	ESDR_WY	ED	4740	0.228	6.88e-57	6.88e-56
3	ESDR_WY	PAFRAC	4740	0.249	2.56e-38	1.71e-37
4	ESDR_CS	ED	1663	-0.209	6.87e-18	2.75e-17
4	ESDR_CS	LPI	1663	0.378	1.02e-57	2.03e-56
4	ESDR_FP	AI	1663	0.239	4.39e-23	2.93e-22
4	ESDR_FP	ED	1663	-0.231	1.23e-21	6.15e-21
4	ESDR_FP	LPI	1663	0.346	5.11e-48	5.11e-47
5	ESDR_SC	ED	92	0.46	3.88e-06	6.05e-05
5	ESDR_SC	LPI	92	-0.434	1.51e-05	6.05e-05
5	ESDR_WY	AI	92	0.383	1.65e-04	4.40e-04
5	ESDR_WY	ED	92	0.438	1.26e-05	6.05e-05
5	ESDR_WY	LPI	92	-0.448	7.56e-06	6.05e-05
5	ESDR_WY	PD	92	-0.402	7.11e-05	2.28e-04
6	ESDR_CS	AI	1415	-0.214	3.96e-16	1.32e-15
6	ESDR_CS	ED	1415	0.212	8.87e-16	2.54e-15
6	ESDR_CS	LPI	1415	-0.269	6.53e-25	3.26e-24
6	ESDR_WY	AI	1415	-0.461	2.90e-75	5.79e-74
6	ESDR_WY	ED	1415	0.418	7.30e-61	7.30e-60

6	ESDR_WY	LPI	1415	-0.235	3.83e-19	1.53e-18
6	ESDR_WY	PAFRAC	1415	0.243	1.52e-05	3.03e-05
6	ESDR_WY	PD	1415	0.297	3.29e-30	2.19e-29
7	ESDR_CS	LPI	3366	0.251	1.31e-49	2.62e-48
7	ESDR_SC	AI	3366	-0.226	2.82e-40	2.82e-39
7	ESDR_SC	PAFRAC	3366	0.216	4.53e-04	6.47e-04
7	ESDR_WY	AI	3366	-0.217	2.52e-37	1.68e-36
7	ESDR_WY	PAFRAC	3366	0.219	3.76e-04	5.79e-04

Note: n is the within-unit sample size; ρ is Spearman's rank correlation; p_raw is the nominal two-sided p-value; p_fdr_BH is the BH–FDR–adjusted p-value.

Table S10. Candidate configuration–CESDR pairs retained for threshold identification after Spearman screening with BH–FDR control (inter-cluster).

Cluster	Response	Landscape	n	Spearman_r	p_raw	p_fdr_BH
1	CESDR	AI	1759	0.326	8.91e-45	1.11e-44
1	CESDR	ED	1759	-0.386	1.68e-63	4.20e-63
1	CESDR	LPI	1759	0.43	4.11e-80	2.06e-79
1	CESDR	PD	1759	-0.341	3.95e-49	6.59e-49
3	CESDR	PAFRAC	4740	0.201	1.90e-25	9.48e-25
4	CESDR	LPI	1663	0.367	3.50e-54	1.75e-53
5	CESDR	ED	92	0.478	1.47e-06	2.95e-06
5	CESDR	LPI	92	-0.483	1.11e-06	2.95e-06
5	CESDR	PD	92	-0.245	1.87e-02	2.49e-02
6	CESDR	AI	1415	-0.275	6.24e-26	3.12e-25
6	CESDR	ED	1415	0.264	5.36e-24	1.34e-23
6	CESDR	LPI	1415	-0.262	1.16e-23	1.94e-23
7	CESDR	PAFRAC	3366	0.204	9.40e-04	2.35e-03

Note: n is the within-unit sample size; ρ is Spearman's rank correlation; p_raw is the nominal two-sided p-value; p_fdr_BH is the BH–FDR–adjusted p-value.

Table S11. Within-cluster summary of model selection outcomes for threshold identification (counts by cluster).

Cluster	N_enter	N_det	N_fail	N_GAM	N_Seg	N_Quad	DetRate
1	14	14	0	13	0	1	1
2	2	2	0	2	0	0	1
3	4	0	4	0	0	0	0
4	5	4	1	4	0	0	0.8
5	6	1	5	1	0	0	0.167
6	8	4	4	4	0	0	0.5
7	5	3	2	3	0	0	0.6

Note: N_enter is the number of candidate pairs entering threshold identification; N_det is the number of pairs ultimately reported in Table S13 with thresholds detected within the supported range. N_fail = N_enter − N_det. N_GAM, N_Seg, and N_Quad are the counts of best-supported models among the N_det pairs. DetRate = N_det / N_enter.

Table S12. Inter-cluster summary of model selection outcomes for threshold identification (counts by cluster).

Cluster	N_enter	N_det	N_fail	N_GAM	N_Seg	N_Quad	DetRate
1	4	4	0	4	0	0	1
3	1	0	1	0	0	0	0
4	1	1	0	1	0	0	1
5	3	0	3	0	0	0	0
6	3	3	0	3	0	0	1
7	1	0	1	0	0	0	0

Note: N_enter is the number of candidate pairs entering threshold identification; N_det is the number of pairs ultimately reported in Table S14 with thresholds detected within the supported range. N_fail = N_enter − N_det. N_GAM, N_Seg, and N_Quad are the counts of best-supported models among the N_det pairs. DetRate = N_det / N_enter.

Table S13. Robustness and uncertainty of within-cluster thresholds (95% CI and bootstrap success).

Cluster	Resp	Metric	Thresh	CI_L	CI_U	Model	BootSR	nBelow	nAbove
1	ESDR_CS	AI	65.237	62.337	79.122	GAM	1	560	1199
1	ESDR_CS	ED	5.001	3.004	7.579	GAM	1	571	1188
1	ESDR_CS	LPI	47.131	44.161	63.069	GAM	1	494	1265
1	ESDR_CS	PD	0.13	0.07	0.148	GAM	1	1515	244
1	ESDR_FP	AI	62.841	60.273	82.922	Quadratic	1	384	1375
1	ESDR_FP	ED	5.91	3.725	7.897	GAM	1	795	964
1	ESDR_FP	LPI	72.176	48.819	79.253	GAM	1	1314	445
1	ESDR_FP	PD	0.11	0.07	0.147	GAM	1	1330	429
1	ESDR_SC	AI	66.764	61.417	86.653	GAM	1	668	1091
1	ESDR_SC	ED	6.213	3.581	7.332	GAM	1	904	855
1	ESDR_SC	LPI	78.085	48.248	85.684	GAM	1	1415	344
1	ESDR_SC	PD	0.06	0.06	0.13	GAM	1	462	1297
1	ESDR_WY	ED	5.034	2.781	7.315	GAM	0.86	571	1188
1	ESDR_WY	LPI	60.075	49.419	79.098	GAM	0.89	1033	726
2	ESDR_FP	LPI	64.894	39.18	80.429	GAM	0.64	131	39
2	ESDR_WY	ED	3.819	2.565	6.531	GAM	0.66	59	111
4	ESDR_CS	LPI	55.985	47.068	82.323	GAM	0.95	333	1330
4	ESDR_FP	AI	76.356	67.471	83.154	GAM	0.97	930	733
4	ESDR_FP	ED	4.989	3.511	6.601	GAM	0.94	734	929
4	ESDR_FP	LPI	65.131	53.89	86.241	GAM	1	557	1106
5	ESDR_WY	AI	66.834	14.573	74.874	GAM	0.72	58	34
6	ESDR_CS	AI	86.817	64.799	88.102	GAM	0.98	1216	199
6	ESDR_CS	ED	3.119	2.79	7.203	GAM	1	207	1208
6	ESDR_CS	LPI	69.271	60.151	69.83	GAM	1	1137	278
6	ESDR_WY	AI	78.598	67.256	85.772	GAM	0.75	962	453
7	ESDR_CS	LPI	76.322	73.985	91.121	GAM	1	934	2432
7	ESDR_SC	AI	76.276	74.258	93.996	GAM	1	701	2665
7	ESDR_WY	AI	93.996	76.078	96.001	GAM	1	2404	962

Note: Thresh is the breakpoint (x value). CI_L/CI_U are the 2.5th/97.5th percentiles of bootstrap threshold estimates (95% CI) computed only from successful replicates yielding a valid in-range threshold; therefore, the interval is not required to bracket the main-analysis Thresh. BootSR is the success proportion; nBelow/nAbove are sample sizes below/above the threshold. Thresh and CI are rounded to two decimals; ESDR is rounded to three decimals (more digits when |value|<0.01).

Table S14. Robustness and uncertainty of inter-cluster thresholds (95% CI and bootstrap success).

Cluster	Resp	Metric	Thresh	CI_L	CI_U	Model	BootSR	nBelow	nAbove
1	CESDR	AI	86.449	59.517	86.998	GAM	1	1509	250
1	CESDR	ED	5.91	3.654	7.864	GAM	1	795	964
1	CESDR	LPI	77.804	39.229	80.964	GAM	1	1395	364
1	CESDR	PD	0.06	0.06	0.149	GAM	0.99	325	1434
4	CESDR	LPI	71.663	47.055	83.912	GAM	0.99	762	901
6	CESDR	AI	76.665	67.18	86.276	GAM	0.99	888	527
6	CESDR	ED	4.972	3.015	7.204	GAM	1	533	882
6	CESDR	LPI	39.975	40.124	65.812	GAM	1	193	1222

Note: Thresh is the breakpoint (x value). CI_L/CI_U are the 2.5th/97.5th percentiles of bootstrap threshold estimates (95% CI) computed only from successful replicates yielding a valid in-range threshold; therefore, the interval is not required to bracket the main-analysis Thresh. BootSR is the success proportion; nBelow/nAbove are sample sizes below/above the threshold. Thresh and CI are rounded to two decimals; CESDR is rounded to three decimals (more digits when |value|<0.01).

SI References

1. J. Zhang, M. Wang, K. Liu, S. Chen, Z. a. Zhao, Social-ecological system sustainability in China from the perspective of supply-demand balance for ecosystem services. Journal of Cleaner Production 497, 145039 (2025).
2. M. Gao, Y. Hu, X. Liu, M. Liang, Revealing multi-scale characteristics of ecosystem services supply and demand imbalance to enhance refined ecosystem management in China. Ecological Indicators 170, 112971 (2025).
3. P. Huang et al., Integrating supply-demand conflict tradeoff of ecosystem services into multi-scenario land use optimization. Habitat International 167, 103623 (2026).
4. Y. Gao et al., Spatio-Temporal evolution and scenario-based optimization of urban ecosystem services supply and Demand: A block-scale study in Xiamen, China. Ecological Indicators 172, 113289 (2025).
5. P. Tenerelli, C. Püffel, S. Luque, Spatial assessment of aesthetic services in a complex mountain region: combining visual landscape properties with crowdsourced geographic information. Landscape Ecology 32, 1097-1115 (2017).
6. C. Wu, X. Guo, F. Liu, E. Dai, Land use zoning planning based on ecosystem services can improve regional comprehensive benefits. Ecological Indicators 181, 114420 (2025).
7. S. Chen, X. Wang, T. Liu, M. Xie, Q. Lin, Using geo-data and social media images to explore the supply and demand of cultural ecosystem services for terraces in China. Ecosystem Services 76, 101778 (2025).
8. X. Liu et al., Long‐term soil warming changes the profile of primary metabolites in fine roots of Norway spruce in a temperate montane forest. Plant, Cell & Environment 47, 4212-4226 (2024).
9. C. Miti, A. E. Milne, K. E. Giller, R. M. Lark, The concepts and quantification of yield gap using boundary lines. A review. Field Crops Research 311, 109365 (2024).
10. J. Lin et al., Integrating ecosystem services and ecological sensitivity to assess ecological restoration potential and determine thresholds in the Wujiang River Basin, southwest China. Ecological Indicators 179, 114233 (2025).
11. F. J. Richter et al., Effects of management practices on the ecosystem-service multifunctionality of temperate grasslands. Nature communications 15, 3829 (2024).
12. Q. Zheng et al., Drivers and dominant pathways for ecosystem service trade-offs in the Luo River Basin at the local optimal scale. Journal of Environmental Management 398, 128551 (2026).
